# Theoretical and numerical analysis for transmission dynamics of COVID-19 mathematical model involving Caputo–Fabrizio derivative

**DOI:** 10.1186/s13662-021-03316-w

**Published:** 2021-03-24

**Authors:** Sabri T. M. Thabet, Mohammed S. Abdo, Kamal Shah

**Affiliations:** 1 Department of Mathematics, University of Aden, Aden, Yemen; 2 Department of Mathematics, Hodeidah University, Hodeidah, Yemen; 3 Department of Mathematics, University of Malakand, Chakdara, Dir(L), KPK Pakistan

**Keywords:** 34A08, 97M70, COVID-19, Caputo–Fabrizio derivative, Picard’s iterative method, Laplace transform, Adomian decomposition method

## Abstract

This manuscript is devoted to a study of the existence and uniqueness of solutions to a mathematical model addressing the transmission dynamics of the coronavirus-19 infectious disease (COVID-19). The mentioned model is considered with a nonsingular kernel type derivative given by Caputo–Fabrizo with fractional order. For the required results of the existence and uniqueness of solution to the proposed model, Picard’s iterative method is applied. Furthermore, to investigate approximate solutions to the proposed model, we utilize the Laplace transform and Adomian’s decomposition (LADM). Some graphical presentations are given for different fractional orders for various compartments of the model under consideration.

## Introduction

COVID-19 is the nuisance word today that affected individuals all over the world as it marched forward across continents and spread throughout all countries. Coronavirus caused a total loss of motion of the development on the planet, which hit the economy and mended nature. It has made dread, despondency and uneasiness in individuals with social separating and the main treatment has become face cover, hand washing, and chemicals that are delivered on the basis of an everyday schedule. Governments stand weak in the face of startlingly high death rates particularly with the old and individuals who have reactions from interminable illnesses, the clinical community addressing patients in danger, made researchers search for an antibody and specialists develop scientific models to restrict the spread of this fatal infection.

The historical backdrop of coronaviruses started in 1965 [1]. It was found in the cultures of the fetal trachea organs obtained from the respiratory system of an adult suffering from a common cold. Recent research has revealed that coronary respiratory infections frequently emerge in the spring and winter more than in the autumn and summer seasons. COVID-19 added up to 35 of overall respiratory viral vigor through epidemics.

A mathematical modeling of disease transmission is intended to understand the dynamic spread of infection between individuals; see [2–6] and the references therein. Actually, it has been observed that infectious disease models have many advantages for forestalling and pointing out rising infectious diseases as well as controlling them like COVID-19. The spreading of infections was found to follow an exponential function and the growth rate differs from 2 to 5 days in some regions. According to the latest statistics to the date of July-01-2020, confirmed infections with the coronavirus have exceeded 10,609,665 worldwide, while the number of deaths has reached 514,449, and the number of people recovered has risen to 5,817,869, according to the Worldometer website that specializes in counting COVID-19 victims.

Infectious sicknesses represent a major threat to people, and additionally to the nation’s economy. A careful comprehension of the behavior of diseases plays a noteworthy role in the decline of the infection in the population. Execution of an advantageous technique contra ailment transportation is another challenge. Various disease models were created in the recent literature that permit us to more readily control the spread of the infection. The vast majority of these models are established by ordinary differential equations; see [7–13]. Nonetheless, as of lately the job of fractional calculus that deals with fractional orders has shown up, as it has a noticeable job in the understanding of real-world phenomena, just as in demonstrating modeling of the exact description of hereditary qualities and memory [14–17]. As of lately, it has been seen that fractional differential equations can be employed to model worldwide problems with more subtlety; see [18, 19].

Recently, new fractional operators were developed that give a precise description of memory and have succeeded with regards to modeling infectious ailments; see [20–22]. The worldwide issue of the spread of the ailment attracted the consideration of analysts from different fields, which prompted the rise of various propositions to examine and predict the development of the epidemic [23, 24]. Our contribution relates to the consideration of the best-known class, which is the classification that presently shows up in clinical diaries [25–36]. This new work incorporates various theoretical and practical analyses on examining the dynamic conduct of the speed of spreading the coronavirus infection (COVID-19) disease and how to lessen the spread of infection in the public arena, and numerical simulations are likewise considered in this work.

As is well known, fractional calculus has a vast range of applicability in expounding convoluted dynamical systems with memory effects in different areas of engineering, biological sciences, and social sciences. Furthermore, the fractional order derivatives given by Caputo and Riemann–Liouville contain a singular type kernel. Fractional differential operators in fact are definite integrals which geometrically represent a complete spectrum of the functions or accumulation. The singular kernel some time creates difficulty during numerical analysis. This is because of its local singular kernel. To overcome this difficulty, Caputo and Fabrizo in 2016 introduced the concept of a nonlocal nonsingular kernel type derivative; see [37]. Recently many authors have proved that the mentioned derivative has interesting features in the descriptions of many processes and phenomena in the thermal sciences; see [38–42]. Keeping the importance of fractional derivatives, recently authors have investigated some models of COVID-19 from different aspects; see [43, 44]. Very recently, authors [45] studied the behavior of COVID-19 transmission through new control strategies, involving all possible conditions of human-to-human transmission.

Mathematical models of infectious disease under fractional order derivatives provide a comprehensive description of the global and local dynamics. Furthermore, such a kind of models in which fractional calculus is involved more precisely describes the phenomena in the best way. Motivated by [37] and [45], we will study the following COVID-19 model with the Caputo–Fabrizio fractional derivative:

1.1 $$ \textstyle\begin{cases} {}^{CF}\mathbb{D}^{\theta }\mathcal{E}(t)=\mathcal{B}-\alpha _{1} \mathcal{E}\mathcal{I}+\alpha _{7}\mathcal{E}\mathcal{D}+\alpha _{9} \mathcal{H}+\alpha _{10}\mathcal{E}\mathcal{I}-\delta \mathcal{E}, \\ {}^{CF}\mathbb{D}^{\theta }\mathcal{I}(t)=\alpha _{1}\mathcal{E} \mathcal{I}-\alpha _{2}\mathcal{I}-\alpha _{6}\mathcal{I}-\alpha _{8} \mathcal{I}-\alpha _{10}\mathcal{E}\mathcal{I}-\delta \mathcal{I}, \\ {}^{CF}\mathbb{D}^{\theta }\mathcal{C}(t)=\alpha _{2}\mathcal{I}- \alpha _{5}\mathcal{C}-\alpha _{3}\mathcal{C}+\alpha _{4}\mathcal{H}- \delta \mathcal{C} , \\ {}^{CF}\mathbb{D}^{\theta }\mathcal{H}(t)=\alpha _{3}\mathcal{C}- \alpha _{4}\mathcal{H}+\alpha _{8}\mathcal{I}-\alpha _{9}\mathcal{H}- \delta \mathcal{H}, \\ {}^{CF}\mathbb{D}^{\theta }\mathcal{D}(t)=\alpha _{5}\mathcal{C}+ \alpha _{6}\mathcal{I}-\alpha _{7}\mathcal{E}\mathcal{D}, \end{cases} $$

with the initial conditions

1.2 $$ \textstyle\begin{cases} \mathcal{E}(0)=\mathcal{E}_{0}\geq 0,\qquad \mathcal{I}(0)= \mathcal{I}_{0} \geq 0,\qquad \mathcal{C}(0)=\mathcal{C}_{0}\geq 0, \\ \mathcal{H}(0)= \mathcal{H}_{0}\geq 0, \qquad \mathcal{D}(0)=\mathcal{D}_{0} \geq 0, \end{cases} $$

where $0\leq t \leq T <\infty $ and ${}^{CF}\mathcal{D}^{\theta }$ denotes the Caputo–Fabrizio fractional derivative of order $0<\theta \leq 1 $. The details of the given model are described as follows:

- The total population is classified into five compartments of individuals as follows: $\mathcal{E}(t)$ is for exposed (uninfected but surrounded by infection); $\mathcal{I}(t)$ for infected (with obvious clinical symptoms but not critical); $\mathcal{C}(t)$ for critically infected; $\mathcal{H}(t)$ for hospitalized individuals; $\mathcal{D}(t)$ for dead individuals due to COVID-19.
- $\mathcal{B}$ represents a birth rate of exposed individuals.
- *δ* is the rate of natural mortality.
- $\alpha _{1}$ is the rate of individuals transmission from exposed to infected compartment.
- $\alpha _{2}$ is the rate of critical cases of infected individuals.
- $\alpha _{3}$ is the rate of critical infected hospitalized.
- $\alpha _{4}$ is the rate of hospitalized individuals which not recovered and stay in critical case.
- $\alpha _{5}$ is the rate of death in critically infected individuals class.
- $\alpha _{6}$ is the rate of death in infected individuals class.
- $\alpha _{7}$ is the rate of infected people due to spreading infection from a dead body.
- $\alpha _{8}$ is the rate of infected individuals which hospitalized without passing in critical case.
- $\alpha _{9}$ is the rate of recovered which individuals hospitalized and get exposed again.
- $\alpha _{10}$ is the rate of recovered in infected individuals class due to powerful immunity and get exposed again.

We first establish the existence theory of the model under the said derivative via a Picard type analysis of fixed point theory. Then on using LADM, we derive a semi-analytical solution to the problem under consideration. Here we remark that treating Caputo–Fabrizo type differential equations by LADM is very rare in the literature.

Our manuscript is arranged as follows. Some useful fundamentals are given in Sect. 2. Further theoretical results are given in Sect. 3. Numerical results are presented in Sect. 4. Finally, a brief conclusion is given in Sect. 5.

## Preliminaries

In this section, we recall some useful fundamentals related to fractional calculus.

### Definition 2.1

([46])

The Caputo–Fabrizio fractional derivative of order $\gamma \in (0,1) $ for a function $\varOmega \in \mathcal{H}^{1}(a,b)$ is given by

$$ {}^{CF}\mathcal{D}^{\gamma }\varOmega (t)= \frac{(2-\gamma )\mathcal{N}(\gamma )}{ 2(1-\gamma ) }\int _{a}^{t}\exp \biggl( \frac{-\gamma }{1-\gamma }(t-s ) \biggr) \varOmega ^{\prime }(s )\,ds ,\quad t>0, $$

where $\mathcal{N}(\gamma )$ is the normalization function which defined by $\mathcal{N}(\gamma )=\frac{2}{2-\gamma }$ and it satisfies $\mathcal{N}(0)=\mathcal{N}(1)=1$. If $\varOmega \notin \mathcal{H}^{1}(a,b)$, then the derivative can be represented for $\varOmega \in \mathcal{L}^{1}(-\infty ,b)$ as

$$ {}^{CF}\mathcal{D}^{\gamma }\varOmega (t)= \frac{\gamma \mathcal{N}(\gamma )}{ 1-\gamma }\int _{-\infty }^{b} \bigl(\varOmega (t)-\varOmega (s) \bigr) \exp \biggl( \frac{-\gamma }{1-\gamma }(t-s ) \biggr)\,ds. $$

### Definition 2.2

([46])

The Caputo–Fabrizio fractional integral of order $\gamma \in (0,1] $ for a function $\varOmega \in \mathcal{H}^{1}(0,T)$ is given by

$$ {}^{CF}\mathcal{I}^{\gamma }\varOmega (t)= \frac{2(1-\gamma ) }{(2-\gamma )\mathcal{N}(\gamma )}\varOmega (t)+\frac{2\gamma }{(2-\gamma )\mathcal{N}(\gamma )} \int _{0}^{t} \varOmega (s )\,ds ,\quad t\geq 0. $$

### Lemma 2.1

([46])

*The solution of the following system*:

$$ \textstyle\begin{cases} {}^{CF}\mathcal{D}^{\gamma }\varOmega (t) =\psi (t), \quad \gamma \in (0,1], \\ \varOmega (0) =\varOmega _{0}\in \mathbb{R}, \end{cases} $$

*is given by*

$$ \varOmega (t)=\varOmega _{0}+ \frac{2(1-\gamma ) }{(2-\gamma )\mathcal{N}(\gamma )} \bigl[\psi (t)- \psi (0) \bigr]+ \frac{2\gamma }{(2-\gamma )\mathcal{N}(\gamma )} \int _{0}^{t}\psi (s )\,ds . $$

### Lemma 2.2

([46])

*The Laplace transform of fractional derivative in the sense of Caputo–Fabrizio of order* $\gamma \in (0,1] $ *for a function* $\varOmega (t)$ *is defined as follows*:

$$ \mathcal{L} \bigl[ {}^{CF}\mathbb{D}^{\gamma }\varOmega (t) \bigr] =\frac{ s \mathcal{L} [ \varOmega (t) ] -\varOmega (0)}{s+\gamma (1-s) },\quad s\geq 0.$$

## Theoretical approach

In this section, we aim to present the existence and uniqueness result for a solution of the model (1.1)–(1.2) by using Picard’s successive iterative approximation method [47]. For this purpose, let $X=\Delta \times \Delta \times \Delta \times \Delta \times \Delta $ denote a Banach space with supremum norm

$$\begin{aligned} \Vert \mathcal{X} \Vert =& \bigl\Vert (\mathcal{E},\mathcal{I}, \mathcal{C}, \mathcal{H}, \mathcal{D}) \bigr\Vert \\ =&\sup_{t\in [0,T]}\bigl\{ \mathcal{E}(t)+\mathcal{I}(t)+ \mathcal{C}(t)+ \mathcal{H}(t)+ \mathcal{D}(t)\bigr\} , \mathcal{E}, \mathcal{I}, \mathcal{C}, \mathcal{H}, \mathcal{D}\in \Delta =C[0,T]. \end{aligned}$$

Now, we rewrite the model (1.1) in the following form:

3.1 $$ \textstyle\begin{cases} {}^{CF}\mathbb{D}^{\theta }\mathcal{E}=\mathcal{X}_{1}(t, \mathcal{E}, \mathcal{I}, \mathcal{C}, \mathcal{H}, \mathcal{D}), \\ {}^{CF}\mathbb{D}^{\theta }\mathcal{I}=\mathcal{X}_{2}(t, \mathcal{E}, \mathcal{I}, \mathcal{C}, \mathcal{H}, \mathcal{D}), \\ {}^{CF}\mathbb{D}^{\theta }\mathcal{C}=\mathcal{X}_{3}(t, \mathcal{E}, \mathcal{I}, \mathcal{C}, \mathcal{H}, \mathcal{D}), \\ {}^{CF}\mathbb{D}^{\theta }\mathcal{H}=\mathcal{X}_{4}(t, \mathcal{E}, \mathcal{I}, \mathcal{C}, \mathcal{H}, \mathcal{D}), \\ {}^{CF}\mathbb{D}^{\theta }\mathcal{D}=\mathcal{X}_{5}(t, \mathcal{E}, \mathcal{I}, \mathcal{C}, \mathcal{H}, \mathcal{D}), \end{cases} $$

where

3.2 $$ \textstyle\begin{cases} \mathcal{X}_{1}(t, \mathcal{E}, \mathcal{I}, \mathcal{C}, \mathcal{H}, \mathcal{D})=\mathcal{B}-\alpha _{1}\mathcal{E}\mathcal{I}+\alpha _{7} \mathcal{E}\mathcal{D}+\alpha _{9}\mathcal{H}+\alpha _{10}\mathcal{E} \mathcal{I}-\delta \mathcal{E}, \\ \mathcal{X}_{2}(t, \mathcal{E}, \mathcal{I}, \mathcal{C}, \mathcal{H}, \mathcal{D})=\alpha _{1}\mathcal{E}\mathcal{I}-\alpha _{2}\mathcal{I}- \alpha _{6}\mathcal{I}-\alpha _{8}\mathcal{I}-\alpha _{10}\mathcal{E} \mathcal{I}-\delta \mathcal{I}, \\ \mathcal{X}_{3}(t, \mathcal{E}, \mathcal{I}, \mathcal{C}, \mathcal{H}, \mathcal{D})=\alpha _{2}\mathcal{I}-\alpha _{5}\mathcal{C}-\alpha _{3} \mathcal{C}+\alpha _{4}\mathcal{H}-\delta \mathcal{C}, \\ \mathcal{X}_{4}(t, \mathcal{E}, \mathcal{I}, \mathcal{C}, \mathcal{H}, \mathcal{D})=\alpha _{3}\mathcal{C}-\alpha _{4}\mathcal{H}+\alpha _{8} \mathcal{I}-\alpha _{9}\mathcal{H}-\delta \mathcal{H}, \\ \mathcal{X}_{5}(t, \mathcal{E}, \mathcal{I}, \mathcal{C}, \mathcal{H}, \mathcal{D})= \alpha _{5}\mathcal{C}+\alpha _{6}\mathcal{I}-\alpha _{7} \mathcal{E}\mathcal{D}. \end{cases} $$

Using (3.1) and (3.2), our model (1.1)–(1.2) becomes

3.3 $$ \textstyle\begin{cases} {}^{CF}\mathbb{D}^{\theta }\Upsilon (t)=\Psi (t,\Upsilon (t)),\quad t\in [0,T], \\ \Upsilon (0)=\Upsilon _{0}\geq 0, \end{cases} $$

such that

3.4 $$ϒ(t)=\left[\begin{array}{c} E(t)\\ I(t)\\ C(t)\\ H(t)\\ D(t) \end{array}\right],\;{ϒ}_{0}(t)=\left[\begin{array}{c} E_{0}\\ I_{0}\\ C_{0}\\ H_{0}\\ D_{0} \end{array}\right],\;\Psi \left(t,ϒ(t)\right)=\left[\begin{array}{c} X_{1}(t,E,I,C,H,D)\\ X_{2}(t,E,I,C,H,D)\\ X_{3}(t,E,I,C,H,D)\\ X_{4}(t,E,I,C,H,D)\\ X_{5}(t,E,I,C,H,D) \end{array}\right],$$

and

3.5 $$\Psi_{0}(t)=\left[\begin{array}{c} X_{1}(0,E_{0},I_{0},C_{0},H_{0},D_{0})\\ X_{2}(0,E_{0},I_{0},C_{0},H_{0},D_{0})\\ X_{3}(0,E_{0},I_{0},C_{0},H_{0},D_{0})\\ X_{4}(0,E_{0},I_{0},C_{0},H_{0},D_{0})\\ X_{5}(0,E_{0},I_{0},C_{0},H_{0},D_{0}) \end{array}\right].$$

According to Lemma 2.1, the system (3.3) is equivalent to the following fractional integral equation:

3.6 $$ \Upsilon (t)=\Upsilon _{0}+ \frac{2(1-\theta ) }{(2-\theta )\mathcal{N}(\theta )} \bigl[\Psi \bigl(t, \Upsilon (t)\bigr)-\Psi _{0} \bigr]+ \frac{2\theta }{(2-\theta )\mathcal{N}(\theta )} \int _{0}^{t}\Psi \bigl(s, \Upsilon (s) \bigr) \,ds . $$

### Theorem 3.1

*Let* $\Psi \in X $ *be a continuous function*. *Suppose there exists a constant* $\ell >0 $ *such that* $\vert \Psi (t,\Upsilon _{1} )-\Psi (t,\Upsilon _{2} ) \vert \leq \ell \vert \Upsilon _{1}-\Upsilon _{2} \vert $, *for all* $t\in [0,T]$, $\Upsilon _{1}, \Upsilon _{2}\in X$, *and there exists a positive constant* *M* *such that* $\sup_{t\in [0,T]} \vert \Psi (t,\Upsilon _{0}(t)) \vert \leq M$. *Then there exists a unique solution* $\Upsilon (t)$ *for the model* (1.1)*–*(1.2) *on* $[0, T]$, *provided that*

3.7 $$ \ell \biggl[\frac{2(1-\theta ) }{(2-\theta )\mathcal{N}(\theta )}+ \frac{2\theta T}{(2-\theta )\mathcal{N}(\theta )} \biggr] < 1. $$

### Proof

Obviously, the solution of the model (1.1)–(1.2) is equivalent to the fractional integral equation (3.6). Consider

3.8 $$ \Upsilon _{0} (t)=\Upsilon _{0}- \frac{2(1-\theta ) }{(2-\theta )\mathcal{N}(\theta )} \Psi _{0} $$

and

3.9 $$\begin{aligned} \Upsilon _{n} (t) =&\Upsilon _{0}- \frac{2(1-\theta ) }{(2-\theta )\mathcal{N}(\theta )}\Psi _{0}+ \frac{2(1-\theta ) }{(2-\theta )\mathcal{N}(\theta )}\Psi \bigl(t, \Upsilon _{n-1}(t)\bigr) \\ &{}+ \frac{ 2\theta }{(2-\theta )\mathcal{N}(\theta )} \int _{0}^{t}\Psi \bigl(s, \Upsilon _{n-1}(s) \bigr)\,ds . \end{aligned}$$

Its clear that $\Upsilon _{n} (t)=\Upsilon _{0}+ \sum_{i=1}^{n} (\Upsilon _{i}(t)- \Upsilon _{i-1}(t) )$, which is a partial sum of $\Upsilon _{0}+ \sum_{i=1}^{\infty } (\Upsilon _{i}(t)-\Upsilon _{i-1}(t) )$. Our target is a proof that the sequence $\{\Upsilon _{n}(t)\}$ converges to $\Upsilon (t)$.

Now, by mathematical induction, for each $t\in [0,T]$, we prove that

3.10 $$ \Vert \Upsilon _{n}-\Upsilon _{n-1} \Vert \leq M\ell ^{n-1} \biggl[\frac{2(1-\theta ) }{(2-\theta )\mathcal{N}(\theta )}+ \frac{2\theta T}{(2-\theta )\mathcal{N}(\theta )} \biggr]^{n}, \quad n\in \mathbb{N}. $$

From Eqs. (3.8) and (3.9), we get

$$\begin{aligned} \Vert \Upsilon _{1}-\Upsilon _{0} \Vert &=\sup _{t\in [0,T]} \biggl\vert \frac{2(1-\theta ) }{(2-\theta )\mathcal{N}(\theta )} \Psi \bigl(t, \Upsilon _{0}(t)\bigr)+ \frac{2 \theta }{(2-\theta )\mathcal{N}(\theta )} \int _{0}^{t}\Psi \bigl(s, \Upsilon _{0}(s) \bigr)\,ds \biggr\vert \\ &\leq \frac{2(1-\theta ) }{(2-\theta )\mathcal{N}(\theta )}\sup_{t \in [0,T]} \bigl\vert \Psi \bigl(t,\Upsilon _{0}(t)\bigr) \bigr\vert + \frac{2\theta }{(2-\theta )\mathcal{N}(\theta )} \int _{0}^{t}\sup_{t\in [0,T]} \bigl\vert \Psi \bigl(s,\Upsilon _{0}(s) \bigr) \bigr\vert \,ds \\ &\leq \frac{2M(1-\theta ) }{(2-\theta )\mathcal{N}(\theta )}+ \frac{2M\theta T}{(2-\theta )\mathcal{N}(\theta )} . \end{aligned}$$

Thus, the inequality (3.10) is true for $n=1$. Next, we suppose that the inequality (3.10) holds for $n = k$. Then

$$\begin{aligned} \Vert \Upsilon _{k+1}-\Upsilon _{k} \Vert ={}&\sup _{t\in [0,T]} \biggl\vert \frac{2(1-\theta ) }{(2-\theta )\mathcal{N}(\theta )} \Psi \bigl(t, \Upsilon _{k}(t)\bigr)+ \frac{2\theta }{(2-\theta )\mathcal{N}(\theta )} \int _{0}^{t}\Psi \bigl(s, \Upsilon _{k}(s) \bigr)\,ds \\ &{} -\frac{2(1-\theta ) }{(2-\theta )\mathcal{N}(\theta )} \Psi \bigl(t,\Upsilon _{k-1}(t)\bigr)- \frac{2\theta }{(2-\theta )\mathcal{N}(\theta )} \int _{0}^{t}\Psi \bigl(s, \Upsilon _{k-1}(s) \bigr)\,ds\biggr\vert \\ \leq{}& \frac{2(1-\theta ) }{(2-\theta )\mathcal{N}(\theta )}\sup_{t \in [0,T]} \bigl\vert \Psi \bigl(t,\Upsilon _{k}(t)\bigr)-\Psi \bigl(t,\Upsilon _{k-1}(t)\bigr) \bigr\vert \\ & {} + \frac{2\theta }{(2-\theta )\mathcal{N}(\theta )} \int _{0}^{t}\sup_{t\in [0,T]} \bigl\vert \Psi \bigl(s,\Upsilon _{k}(s) \bigr)-\Psi \bigl(s, \Upsilon _{k-1}(s) \bigr) \bigr\vert \,ds \\ \leq{}& \frac{2\ell (1-\theta ) }{(2-\theta )\mathcal{N}(\theta )} \Vert \Upsilon _{k}-\Upsilon _{k-1} \Vert + \frac{2\ell \theta }{(2-\theta )\mathcal{N}(\theta )} \int _{0}^{t} \Vert \Upsilon _{k}-\Upsilon _{k-1} \Vert \,ds \\ \leq{}& \frac{2\ell (1-\theta ) }{(2-\theta )\mathcal{N}(\theta )}M \ell ^{k-1} \biggl[ \frac{2(1-\theta ) }{(2-\theta )\mathcal{N}(\theta )}+ \frac{2\theta T}{(2-\theta )\mathcal{N}(\theta )} \biggr]^{k} \\ & {} + \frac{2\ell \theta }{(2-\theta )\mathcal{N}(\theta )} \int _{0}^{t} M\ell ^{k-1} \biggl[ \frac{2(1-\theta ) }{(2-\theta )\mathcal{N}(\theta )}+ \frac{2\theta T}{(2-\theta )\mathcal{N}(\theta )} \biggr]^{k}\,ds \\ \leq{}& M\ell ^{(k+1)-1} \biggl[ \frac{2(1-\theta ) }{(2-\theta )\mathcal{N}(\theta )}+ \frac{2\theta T}{(2-\theta )\mathcal{N}(\theta )} \biggr]^{(k+1)}. \end{aligned}$$

So, the inequality (3.10) is true for $n=k+1$. Hence, by the principle of mathematical induction the inequality (3.10) is satisfied for each $n\in \mathbb{N}$ and each $t\in [0,T]$. Therefore, we have

3.11 $$ \sum_{n=1}^{\infty } \Vert \Upsilon _{n}-\Upsilon _{n-1} \Vert \leq \sum _{n=1}^{\infty } M\ell ^{n-1} \biggl[ \frac{2(1-\theta ) }{(2-\theta )\mathcal{N}(\theta )}+ \frac{2\theta T}{(2-\theta )\mathcal{N}(\theta )} \biggr]^{n}. $$

By the condition (3.7), the geometric series in the right hand side of the above inequality is convergent and by the comparison test the series $\sum_{n=1}^{\infty } \Vert \Upsilon _{n}-\Upsilon _{n-1} \Vert $ also is convergent, which shows that $\Upsilon _{0}+\sum_{n=1}^{\infty } \Vert \Upsilon _{n}- \Upsilon _{n-1} \Vert $ converges. Let us suppose

$$ \Upsilon =\Upsilon _{0}+\sum_{n=1}^{\infty } \Vert \Upsilon _{n}- \Upsilon _{n-1} \Vert . $$

Thus,

3.12 $$ \Vert \Upsilon _{n}-\Upsilon \Vert \longrightarrow 0 \quad \mbox{as } n\longrightarrow \infty . $$

This proves that the solution of proposed model exists. Actually, by using (3.12), we get

$$\begin{aligned} \bigl\Vert \Psi \bigl(\cdot ,\Upsilon _{n-1}(\cdot )\bigr)-\Psi \bigl(\cdot ,\Upsilon (\cdot )\bigr) \bigr\Vert \leq \ell \Vert \Upsilon _{n-1}-\Upsilon \Vert \longrightarrow 0 \quad \mbox{as } n \longrightarrow \infty . \end{aligned}$$

So,

3.13 $$\begin{aligned} \bigl\Vert \Psi \bigl(\cdot ,\Upsilon _{n-1}(\cdot )\bigr)-\Psi \bigl(\cdot ,\Upsilon (\cdot )\bigr) \bigr\Vert \longrightarrow 0 \quad \mbox{as } n\longrightarrow \infty . \end{aligned}$$

Hence, taking the limit $n\longrightarrow \infty $ on both sides of (3.9) and using (3.13), we conclude

3.14 $$\begin{aligned} \Upsilon (t) =&\Upsilon _{0}- \frac{2(1-\theta ) }{(2-\theta )\mathcal{N}(\theta )}\Psi _{0}+ \frac{2(1-\theta ) }{(2-\theta )\mathcal{N}(\theta )}\Psi \bigl(t, \Upsilon (t)\bigr) \\ &{}+ \frac{ 2\theta }{(2-\theta )\mathcal{N}(\theta )} \int _{0}^{t}\Psi \bigl(s, \Upsilon (s) \bigr) \,ds , \end{aligned}$$

which is the solution of the model (1.1)–(1.2).

Finally, we show the solution ϒ is unique. To this aim, let ϒ̃ be another solution of our model. Then we get

$$\begin{aligned} \Vert \Upsilon -\tilde{\Upsilon } \Vert \leq{}& \frac{2(1-\theta ) }{(2-\theta )\mathcal{N}(\theta )} \sup _{t\in [0,T]} \bigl\vert \Psi \bigl(t,\Upsilon (t)\bigr)-\Psi \bigl(t,\tilde{\Upsilon }(t)\bigr) \bigr\vert \\ &{} + \frac{2\theta }{(2-\theta )\mathcal{N}(\theta )} \int _{0}^{t}\sup_{t\in [0,T]} \bigl\vert \Psi \bigl(s,\Upsilon (s) \bigr)-\Psi \bigl(s,\tilde{\Upsilon }(s) \bigr) \bigr\vert \,ds \\ \leq{}& \frac{2\ell (1-\theta ) }{(2-\theta )\mathcal{N}(\theta )} \Vert \Upsilon -\tilde{\Upsilon } \Vert + \frac{2 \ell \theta }{(2-\theta )\mathcal{N}(\theta )} \int _{0}^{t} \Vert \Upsilon -\tilde{\Upsilon } \Vert \,ds \\ \leq{}& \ell \biggl[ \frac{2(1-\theta ) }{(2-\theta )\mathcal{N}(\theta )}+ \frac{2\theta T}{(2-\theta )\mathcal{N}(\theta )} \biggr] \Vert \Upsilon -\tilde{\Upsilon } \Vert . \end{aligned}$$

Hence, in view of condition (3.7), we should have $\Vert \Upsilon -\tilde{\Upsilon }\Vert =0$, thus $\Upsilon (t)=\tilde{\Upsilon }(t)$. This finishes the proof. □

## Numerical approach

Throughout this section, we introduce the series type solution of the proposed model (1.1)–(1.2), by utilizing the Laplace transform with the Adomian decomposition method [48]. For the convergence of such a method, we refer the reader to [49]. Applying the Laplace transform to both sides of (1.1), we get

4.1 $$ \textstyle\begin{cases} \mathcal{L}[\mathcal{E}(t)]=\frac{\mathcal{E}(0)}{s}+ \frac{s+\theta (1-s)}{s}\mathcal{L} [\mathcal{B}-\alpha _{1} \mathcal{E}\mathcal{I}+\alpha _{7}\mathcal{E}\mathcal{D}+\alpha _{9} \mathcal{H}+\alpha _{10}\mathcal{E}\mathcal{I}-\delta \mathcal{E} ], \\ \mathcal{L}[\mathcal{I}(t)]=\frac{\mathcal{I}(0)}{s}+ \frac{s+\theta (1-s)}{s}\mathcal{L} [\alpha _{1}\mathcal{E} \mathcal{I}-\alpha _{2}\mathcal{I}-\alpha _{6}\mathcal{I}-\alpha _{8} \mathcal{I}-\alpha _{10}\mathcal{E}\mathcal{I}-\delta \mathcal{I} ], \\ \mathcal{L}[\mathcal{C}(t)]=\frac{\mathcal{C}(0)}{s}+ \frac{s+\theta (1-s)}{s}\mathcal{L} [\alpha _{2}\mathcal{I}- \alpha _{5}\mathcal{C}-\alpha _{3}\mathcal{C}+\alpha _{4}\mathcal{H}- \delta \mathcal{C} ] , \\ \mathcal{L}[\mathcal{H}(t)]=\frac{\mathcal{H}(0)}{s}+ \frac{s+\theta (1-s)}{s}\mathcal{L} [\alpha _{3}\mathcal{C}- \alpha _{4}\mathcal{H}+\alpha _{8}\mathcal{I}-\alpha _{9}\mathcal{H}- \delta \mathcal{H} ], \\ \mathcal{L}[\mathcal{D}(t)]=\frac{\mathcal{D}(0)}{s}+ \frac{s+\theta (1-s)}{s}\mathcal{L} [\alpha _{5}\mathcal{C}+ \alpha _{6}\mathcal{I}-\alpha _{7}\mathcal{E}\mathcal{D} ]. \end{cases} $$

Next, suppose the solution in the following series type:

4.2 $$ \begin{aligned} &\mathcal{E}(t)=\sum _{n=0}^{\infty }\mathcal{E}_{n}(t), \qquad \mathcal{I}(t)=\sum_{n=0}^{\infty } \mathcal{I}_{n}(t), \qquad \mathcal{C}(t)=\sum _{n=0}^{\infty }\mathcal{C}_{n}(t), \\ &\mathcal{H}(t)=\sum_{n=0}^{\infty } \mathcal{H}_{n}(t),\qquad \mathcal{D}(t)=\sum _{n=0}^{\infty }\mathcal{D}_{n}(t). \end{aligned} $$

Moreover, by Adomian’s polynomial we can decompose the nonlinear terms $\mathcal{E}(t)\mathcal{I}(t)$ and $\mathcal{E}(t)\mathcal{D}(t)$ as follows:

4.3 $$ \mathcal{E}(t)\mathcal{I}(t)=\sum_{n=0}^{\infty }A_{n}( \mathcal{E}, \mathcal{I}),\qquad \mathcal{E}(t)\mathcal{D}(t)=\sum _{n=0}^{\infty }B_{n}( \mathcal{E}, \mathcal{D}), $$

where the Adomian polynomial $A_{n}(\mathcal{E},\mathcal{I})$ can be defined as

4.4 $$ A_{n}(\mathcal{E},\mathcal{I})=\frac{1}{n !} \frac{d^{n}}{d\lambda ^{n}} \Biggl[\sum_{i=0}^{q} \lambda ^{i} \mathcal{E}_{i}(t) \sum _{i=0}^{q}\lambda ^{i} \mathcal{I}_{i}(t) \Biggr]\Bigg|_{\lambda =0} . $$

In particular, we have

4.5 $$ A_{0}(\mathcal{E},\mathcal{I})=\mathcal{E}_{0}(t) \mathcal{I}_{0}(t),\qquad A_{1}(\mathcal{E},\mathcal{I})= \mathcal{E}_{1}(t)\mathcal{I}_{0}(t)+ \mathcal{E}_{0}(t)\mathcal{I}_{1}(t) . $$

Similarly, we can define the polynomial $B_{n}(\mathcal{E},\mathcal{D})$.

Therefore, by applying (4.2)–(4.5) into (4.1), we get

4.6 $$ \textstyle\begin{cases} \mathcal{L}[\sum_{n=0}^{\infty }\mathcal{E}_{n}(t)] \\ \quad = \frac{\mathcal{E}(0)}{s}+\frac{s+\theta (1-s)}{s}\mathcal{L} [ \mathcal{B}-\alpha _{1}\sum_{n=0}^{\infty }A_{n}(\mathcal{E}, \mathcal{I})+\alpha _{7}\sum_{n=0}^{\infty }B_{n}(\mathcal{E}, \mathcal{D}) \\ \qquad {}+\alpha _{9}\sum_{n=0}^{\infty }\mathcal{H}_{n}+\alpha _{10}\sum_{n=0}^{\infty }A_{n}(\mathcal{E},\mathcal{I})- \delta \sum_{n=0}^{\infty }\mathcal{E}_{n} ], \\ \mathcal{L}[\sum_{n=0}^{\infty }\mathcal{I}_{n}(t)] \\ \quad = \frac{\mathcal{I}(0)}{s}+\frac{s+\theta (1-s)}{s}\mathcal{L} [ \alpha _{1}\sum_{n=0}^{\infty }A_{n}(\mathcal{E},\mathcal{I})-\alpha _{2} \sum_{n=0}^{\infty }\mathcal{I}_{n}-\alpha _{6}\sum_{n=0}^{\infty } \mathcal{I}_{n} \\ \qquad {}-\alpha _{8}\sum_{n=0}^{\infty }\mathcal{I}_{n}-\alpha _{10} \sum_{n=0}^{\infty }A_{n}(\mathcal{E},\mathcal{I})- \delta \sum_{n=0}^{\infty }\mathcal{I}_{n} ], \\ \mathcal{L}[\sum_{n=0}^{\infty }\mathcal{C}_{n}(t)] \\ \quad = \frac{\mathcal{C}(0)}{s}+\frac{s+\theta (1-s)}{s}\mathcal{L} [ \alpha _{2}\sum_{n=0}^{\infty }\mathcal{I}_{n}-\alpha _{5}\sum_{n=0}^{ \infty }\mathcal{C}_{n}-\alpha _{3}\sum_{n=0}^{\infty }\mathcal{C}_{n}+ \alpha _{4}\sum_{n=0}^{\infty }\mathcal{H}_{n} \\ \qquad {}-\delta \sum_{n=0}^{\infty }\mathcal{C}_{n} ] , \\ \mathcal{L}[\sum_{n=0}^{\infty }\mathcal{H}_{n}(t)] \\ \quad = \frac{\mathcal{H}(0)}{s}+\frac{s+\theta (1-s)}{s}\mathcal{L} [ \alpha _{3}\sum_{n=0}^{\infty }\mathcal{C}_{n}-\alpha _{4}\sum_{n=0}^{ \infty }\mathcal{H}_{n}+\alpha _{8}\sum_{n=0}^{\infty }\mathcal{I}_{n}- \alpha _{9}\sum_{n=0}^{\infty }\mathcal{H}_{n} \\ \qquad {}-\delta \sum_{n=0}^{\infty }\mathcal{H}_{n} ], \\ \mathcal{L}[\sum_{n=0}^{\infty }\mathcal{D}_{n}(t)] \\ \quad = \frac{\mathcal{D}(0)}{s}+\frac{s+\theta (1-s)}{s}\mathcal{L} [ \alpha _{5}\sum_{n=0}^{\infty }\mathcal{C}_{n}+\alpha _{6}\sum_{n=0}^{ \infty }\mathcal{I}_{n}-\alpha _{7}\sum_{n=0}^{\infty }B_{n}( \mathcal{E},\mathcal{D}) ]. \end{cases} $$

Now, matching the terms on both sides of (4.6), we have

4.7 $$ \textstyle\begin{cases} \mathcal{L}[\mathcal{E}_{0}(t)]=\frac{\mathcal{E}_{0}}{s},\qquad \mathcal{L}[\mathcal{I}_{0}(t)]=\frac{\mathcal{I}_{0}}{s},\qquad \mathcal{L}[\mathcal{C}_{0}(t)]=\frac{\mathcal{C}_{0}}{s},\\ \mathcal{L}[\mathcal{H}_{0}(t)]=\frac{\mathcal{H}_{0}}{s},\qquad \mathcal{L}[\mathcal{D}_{0}(t)]=\frac{\mathcal{D}_{0}}{s}, \\ \mathcal{L}[\mathcal{E}_{1}(t)]=\frac{s+\theta (1-s)}{s}\mathcal{L} [\mathcal{B}-\alpha _{1}A_{0}(\mathcal{E},\mathcal{I})+\alpha _{7} B_{0}(\mathcal{E},\mathcal{D})+\alpha _{9}\mathcal{H}_{0} \\ \hphantom{\mathcal{L}[\mathcal{E}_{1}(t)]=}{}+\alpha _{10} A_{0}(\mathcal{E},\mathcal{I})-\delta \mathcal{E}_{0} ], \\ \mathcal{L}[\mathcal{I}_{1}(t)]=\frac{s+\theta (1-s)}{s}\mathcal{L} [\alpha _{1}A_{0}(\mathcal{E},\mathcal{I})-\alpha _{2}\mathcal{I}_{0}- \alpha _{6}\mathcal{I}_{0}-\alpha _{8}\mathcal{I}_{0} \\ \hphantom{\mathcal{L}[\mathcal{I}_{1}(t)]=}{} -\alpha _{10}A_{0}( \mathcal{E},\mathcal{I})-\delta \mathcal{I}_{0} ], \\ \mathcal{L}[\mathcal{C}_{1}(t)]=\frac{s+\theta (1-s)}{s}\mathcal{L} [\alpha _{2}\mathcal{I}_{0}-\alpha _{5}\mathcal{C}_{0}-\alpha _{3} \mathcal{C}_{0}+\alpha _{4}\mathcal{H}_{0} -\delta \mathcal{C}_{0} ] , \\ \mathcal{L}[\mathcal{H}_{1}(t)]=\frac{s+\theta (1-s)}{s}\mathcal{L} [\alpha _{3}\mathcal{C}_{0}-\alpha _{4}\mathcal{H}_{0}+\alpha _{8} \mathcal{I}_{0}-\alpha _{9}\mathcal{H}_{0} -\delta \mathcal{H}_{0} ], \\ \mathcal{L}[\mathcal{D}_{1}(t)]=\frac{s+\theta (1-s)}{s}\mathcal{L} [\alpha _{5}\mathcal{C}_{0}+\alpha _{6}\mathcal{I}_{0}-\alpha _{7}B_{0}( \mathcal{E},\mathcal{D}) ], \\ \mathcal{L}[\mathcal{E}_{2}(t)]=\frac{s+\theta (1-s)}{s}\mathcal{L} [\mathcal{B}-\alpha _{1}A_{1}(\mathcal{E},\mathcal{I})+\alpha _{7} B_{1}(\mathcal{E},\mathcal{D})+\alpha _{9}\mathcal{H}_{1} \\ \hphantom{\mathcal{L}[\mathcal{E}_{2}(t)]=}{} +\alpha _{10} A_{1}(\mathcal{E},\mathcal{I})-\delta \mathcal{E}_{1} ], \\ \mathcal{L}[\mathcal{I}_{2}(t)]=\frac{s+\theta (1-s)}{s}\mathcal{L} [\alpha _{1}A_{1}(\mathcal{E},\mathcal{I})-\alpha _{2}\mathcal{I}_{1}- \alpha _{6}\mathcal{I}_{1}-\alpha _{8}\mathcal{I}_{1} \\ \hphantom{\mathcal{L}[\mathcal{I}_{2}(t)]=}{} -\alpha _{10}A_{1}( \mathcal{E},\mathcal{I})-\delta \mathcal{I}_{1} ], \\ \mathcal{L}[\mathcal{C}_{2}(t)]=\frac{s+\theta (1-s)}{s}\mathcal{L} [\alpha _{2}\mathcal{I}_{1}-\alpha _{5}\mathcal{C}_{1}-\alpha _{3} \mathcal{C}_{1}+\alpha _{4}\mathcal{H}_{1} -\delta \mathcal{C}_{1} ] , \\ \mathcal{L}[\mathcal{H}_{2}(t)]=\frac{s+\theta (1-s)}{s}\mathcal{L} [\alpha _{3}\mathcal{C}_{1}-\alpha _{4}\mathcal{H}_{1}+\alpha _{8} \mathcal{I}_{1}-\alpha _{9}\mathcal{H}_{1} -\delta \mathcal{H}_{1} ], \\ \mathcal{L}[\mathcal{D}_{2}(t)]=\frac{s+\theta (1-s)}{s}\mathcal{L} [\alpha _{5}\mathcal{C}_{1}+\alpha _{6}\mathcal{I}_{1}-\alpha _{7}B_{1}( \mathcal{E},\mathcal{D}) ], \\ \vdots \\ \mathcal{L}[\mathcal{E}_{n+1}(t)]=\frac{s+\theta (1-s)}{s} \mathcal{L} [\mathcal{B}-\alpha _{1}A_{n}(\mathcal{E},\mathcal{I})+ \alpha _{7} B_{n}(\mathcal{E},\mathcal{D})+\alpha _{9}\mathcal{H}_{n} \\ \hphantom{\mathcal{L}[\mathcal{E}_{n+1}(t)]=}{} + \alpha _{10} A_{n}(\mathcal{E},\mathcal{I})-\delta \mathcal{E}_{n} ], \\ \mathcal{L}[\mathcal{I}_{n+1}(t)]=\frac{s+\theta (1-s)}{s} \mathcal{L} [\alpha _{1}A_{n}(\mathcal{E},\mathcal{I})-\alpha _{2} \mathcal{I}_{n}-\alpha _{6}\mathcal{I}_{n}-\alpha _{8}\mathcal{I}_{n} \\ \hphantom{\mathcal{L}[\mathcal{I}_{n+1}(t)]=}{} - \alpha _{10}A_{n}(\mathcal{E},\mathcal{I})-\delta \mathcal{I}_{n} ], \\ \mathcal{L}[\mathcal{C}_{n+1}(t)]=\frac{s+\theta (1-s)}{s} \mathcal{L} [\alpha _{2}\mathcal{I}_{n}-\alpha _{5}\mathcal{C}_{n}- \alpha _{3}\mathcal{C}_{n}+\alpha _{4}\mathcal{H}_{n} -\delta \mathcal{C}_{n} ] , \\ \mathcal{L}[\mathcal{H}_{n+1}(t)]=\frac{s+\theta (1-s)}{s} \mathcal{L} [\alpha _{3}\mathcal{C}_{n}-\alpha _{4}\mathcal{H}_{n}+ \alpha _{8}\mathcal{I}_{n}-\alpha _{9}\mathcal{H}_{n} -\delta \mathcal{H}_{n} ], \\ \mathcal{L}[\mathcal{D}_{n+1}(t)]=\frac{s+\theta (1-s)}{s} \mathcal{L} [\alpha _{5}\mathcal{C}_{n}+\alpha _{6}\mathcal{I}_{n}- \alpha _{7}B_{n}(\mathcal{E},\mathcal{D}) ],\quad n\geq 0. \end{cases} $$

Next, applying the inverse of a Laplace transform on both sides of (4.7), we get

4.8 $$ \textstyle\begin{cases} \mathcal{E}_{0}(t)=\mathcal{E}_{0}, \qquad \mathcal{I}_{0}(t)=\mathcal{I}_{0},\qquad \mathcal{C}_{0}(t)=\mathcal{C}_{0}, \\ \mathcal{H}_{0}(t)= \mathcal{H}_{0},\qquad \mathcal{D}_{0}(t)=\mathcal{D}_{0}, \\ \mathcal{E}_{1}(t)= [\mathcal{B}-\alpha _{1}\mathcal{E}_{0} \mathcal{I}_{0}+\alpha _{7} \mathcal{E}_{0}\mathcal{D}_{0}+\alpha _{9} \mathcal{H}_{0} +\alpha _{10} \mathcal{E}_{0}\mathcal{I}_{0}-\delta \mathcal{E}_{0} ] (1+\theta (t-1) ), \\ \mathcal{I}_{1}(t)= [\alpha _{1}\mathcal{E}_{0}\mathcal{I}_{0}- \alpha _{2}\mathcal{I}_{0}-\alpha _{6}\mathcal{I}_{0}-\alpha _{8} \mathcal{I}_{0} -\alpha _{10}\mathcal{E}_{0}\mathcal{I}_{0}-\delta \mathcal{I}_{0} ] (1+\theta (t-1) ), \\ \mathcal{C}_{1}(t)= [\alpha _{2}\mathcal{I}_{0}-\alpha _{5} \mathcal{C}_{0}-\alpha _{3}\mathcal{C}_{0}+\alpha _{4}\mathcal{H}_{0} - \delta \mathcal{C}_{0} ] (1+\theta (t-1) ) , \\ \mathcal{H}_{1}(t)= [\alpha _{3}\mathcal{C}_{0}-\alpha _{4} \mathcal{H}_{0}+\alpha _{8}\mathcal{I}_{0}-\alpha _{9}\mathcal{H}_{0} - \delta \mathcal{H}_{0} ] (1+\theta (t-1) ), \\ \mathcal{D}_{1}(t)= [\alpha _{5}\mathcal{C}_{0}+\alpha _{6} \mathcal{I}_{0}-\alpha _{7}\mathcal{E}_{0}\mathcal{D}_{0} ] (1+ \theta (t-1) ), \\ \mathcal{E}_{2}(t)=(1+\theta (t-1))\mathcal{B} + (1+2(t-1)\theta + \frac{1}{2}(2-4t+t^{2})\theta ^{2} ) \\ \hphantom{\mathcal{E}_{2}(t)=}{}\times \{(\alpha _{10}-\alpha _{1})\mathcal{I}_{0} [ \mathcal{B}-\alpha _{1}\mathcal{E}_{0}\mathcal{I}_{0}+\alpha _{7} \mathcal{E}_{0}\mathcal{D}_{0}+\alpha _{9}\mathcal{H}_{0} +\alpha _{10} \mathcal{E}_{0}\mathcal{I}_{0}-\delta \mathcal{E}_{0} ] \\ \hphantom{\mathcal{E}_{2}(t)=}{}+ (\alpha _{10}-\alpha _{1})\mathcal{E}_{0} [\alpha _{1} \mathcal{E}_{0}\mathcal{I}_{0}-\alpha _{2}\mathcal{I}_{0}-\alpha _{6} \mathcal{I}_{0}-\alpha _{8}\mathcal{I}_{0} -\alpha _{10}\mathcal{E}_{0} \mathcal{I}_{0}-\delta \mathcal{I}_{0} ] \\ \hphantom{\mathcal{E}_{2}(t)=}{}+\alpha _{7} \mathcal{E}_{0} [\alpha _{5}\mathcal{C}_{0}+\alpha _{6} \mathcal{I}_{0}-\alpha _{7}\mathcal{E}_{0}\mathcal{D}_{0} ] \\ \hphantom{\mathcal{E}_{2}(t)=}{}+(\alpha _{7} \mathcal{D}_{0}-\delta ) [\mathcal{B}-\alpha _{1} \mathcal{E}_{0}\mathcal{I}_{0}+\alpha _{7} \mathcal{E}_{0}\mathcal{D}_{0}+ \alpha _{9}\mathcal{H}_{0} +\alpha _{10} \mathcal{E}_{0}\mathcal{I}_{0}- \delta \mathcal{E}_{0} ] \\ \hphantom{\mathcal{E}_{2}(t)=}{}+\alpha _{9} [\alpha _{3}\mathcal{C}_{0}-\alpha _{4}\mathcal{H}_{0}+ \alpha _{8}\mathcal{I}_{0}-\alpha _{9}\mathcal{H}_{0} -\delta \mathcal{H}_{0} ] \}, \\ \mathcal{I}_{2}(t)= (1+2(t-1)\theta +\frac{1}{2}(2-4t+t^{2}) \theta ^{2} ) \\ \hphantom{\mathcal{I}_{2}(t)=}{}\times \{ (\alpha _{1}-\alpha _{10})\mathcal{I}_{0} [ \mathcal{B}-\alpha _{1}\mathcal{E}_{0}\mathcal{I}_{0}+\alpha _{7} \mathcal{E}_{0}\mathcal{D}_{0}+\alpha _{9}\mathcal{H}_{0} +\alpha _{10} \mathcal{E}_{0}\mathcal{I}_{0}-\delta \mathcal{E}_{0} ] \\ \hphantom{\mathcal{I}_{2}(t)=}{}+ (\alpha _{1}-\alpha _{10})\mathcal{E}_{0} [\alpha _{1} \mathcal{E}_{0}\mathcal{I}_{0}-\alpha _{2}\mathcal{I}_{0}-\alpha _{6} \mathcal{I}_{0}-\alpha _{8}\mathcal{I}_{0} -\alpha _{10}\mathcal{E}_{0} \mathcal{I}_{0}-\delta \mathcal{I}_{0} ] \\ \hphantom{\mathcal{I}_{2}(t)=}{}-(\alpha _{2}+\alpha _{6}+\alpha _{8}+\delta ) [\alpha _{1} \mathcal{E}_{0}\mathcal{I}_{0}-\alpha _{2}\mathcal{I}_{0}-\alpha _{6} \mathcal{I}_{0}-\alpha _{8}\mathcal{I}_{0} \\ \hphantom{\mathcal{I}_{2}(t)=}{} -\alpha _{10}\mathcal{E}_{0} \mathcal{I}_{0}-\delta \mathcal{I}_{0} ] \}, \\ \mathcal{C}_{2}(t)= (1+2(t-1)\theta +\frac{1}{2}(2-4t+t^{2}) \theta ^{2} ) \\ \hphantom{\mathcal{C}_{2}(t)=}{}\times \{\alpha _{2} [\alpha _{1}\mathcal{E}_{0}\mathcal{I}_{0}- \alpha _{2}\mathcal{I}_{0}-\alpha _{6}\mathcal{I}_{0}-\alpha _{8} \mathcal{I}_{0} -\alpha _{10}\mathcal{E}_{0}\mathcal{I}_{0}-\delta \mathcal{I}_{0} ] \\ \hphantom{\mathcal{C}_{2}(t)=}{}-(\alpha _{5}+\alpha _{3}+\delta ) [\alpha _{2}\mathcal{I}_{0}- \alpha _{5}\mathcal{C}_{0}-\alpha _{3}\mathcal{C}_{0}+\alpha _{4} \mathcal{H}_{0} -\delta \mathcal{C}_{0} ] \\ \hphantom{\mathcal{C}_{2}(t)=}{}+\alpha _{4} [\alpha _{3}\mathcal{C}_{0}-\alpha _{4}\mathcal{H}_{0}+ \alpha _{8}\mathcal{I}_{0}-\alpha _{9}\mathcal{H}_{0} -\delta \mathcal{H}_{0} ] \}, \\ \mathcal{H}_{2}(t)= (1+2(t-1)\theta +\frac{1}{2}(2-4t+t^{2}) \theta ^{2} ) \\ \hphantom{\mathcal{H}_{2}(t)=}{}\times \{\alpha _{3} [ \alpha _{2}\mathcal{I}_{0}- \alpha _{5} \mathcal{C}_{0}- \alpha _{3}\mathcal{C}_{0}+\alpha _{4}\mathcal{H}_{0} -\delta \mathcal{C}_{0} ] \\ \hphantom{\mathcal{H}_{2}(t)=}{}+\alpha _{8} [\alpha _{1}\mathcal{E}_{0}\mathcal{I}_{0}- \alpha _{2} \mathcal{I}_{0}- \alpha _{6}\mathcal{I}_{0}- \alpha _{8}\mathcal{I}_{0} - \alpha _{10}\mathcal{E}_{0}\mathcal{I}_{0}- \delta \mathcal{I}_{0} ] \\ \hphantom{\mathcal{H}_{2}(t)=}{}-(\alpha _{4}+\alpha _{9}+\delta ) [\alpha _{3}\mathcal{C}_{0}- \alpha _{4}\mathcal{H}_{0}+\alpha _{8}\mathcal{I}_{0}-\alpha _{9} \mathcal{H}_{0} -\delta \mathcal{H}_{0} ] \}, \\ \mathcal{D}_{2}(t)= (1+2(t-1)\theta +\frac{1}{2}(2-4t+t^{2}) \theta ^{2} ) \\ \hphantom{\mathcal{D}_{2}(t)=}{}\times \{\alpha _{5} [\alpha _{2}\mathcal{I}_{0}-\alpha _{5} \mathcal{C}_{0}-\alpha _{3}\mathcal{C}_{0}+\alpha _{4}\mathcal{H}_{0} - \delta \mathcal{C}_{0} ] \\ \hphantom{\mathcal{D}_{2}(t)=}{}+\alpha _{6} [\alpha _{1}\mathcal{E}_{0}\mathcal{I}_{0}-\alpha _{2} \mathcal{I}_{0}-\alpha _{6}\mathcal{I}_{0}-\alpha _{8}\mathcal{I}_{0} - \alpha _{10}\mathcal{E}_{0}\mathcal{I}_{0}-\delta \mathcal{I}_{0} ] \\ \hphantom{\mathcal{D}_{2}(t)=}{}-\alpha _{7}\mathcal{E}_{0} [\alpha _{5}\mathcal{C}_{0}+\alpha _{6} \mathcal{I}_{0}-\alpha _{7}\mathcal{E}_{0}\mathcal{D}_{0} ] \\ \hphantom{\mathcal{D}_{2}(t)=}{}-\alpha _{7}\mathcal{D}_{0} [\mathcal{B}-\alpha _{1}\mathcal{E}_{0} \mathcal{I}_{0}+\alpha _{7} \mathcal{E}_{0}\mathcal{D}_{0}+\alpha _{9} \mathcal{H}_{0} +\alpha _{10} \mathcal{E}_{0}\mathcal{I}_{0}-\delta \mathcal{E}_{0} ] \}, \end{cases} $$

and so on. Hence, we obtain the required solution as follows:

$$\begin{aligned} & \mathcal{E}(t)=\mathcal{E}_{0}(t)+\mathcal{E}_{1}(t)+ \mathcal{E}_{2}(t)+ \cdots , \qquad \mathcal{I}(t)= \mathcal{I}_{0}(t)+\mathcal{I}_{1}(t)+ \mathcal{I}_{2}(t)+\cdots , \\ & \mathcal{C}(t)=\mathcal{C}_{0}(t)+\mathcal{C}_{1}(t)+ \mathcal{C}_{2}(t)+ \cdots , \qquad \mathcal{H}(t)= \mathcal{H}_{0}(t)+\mathcal{H}_{1}(t)+ \mathcal{H}_{2}(t)+\cdots , \\ & \mathcal{D}(t)=\mathcal{D}_{0}(t)+\mathcal{D}_{1}(t)+ \mathcal{D}_{2}(t)+ \cdots . \end{aligned}$$

### Numerical simulations and discussion

**Figure 1:**
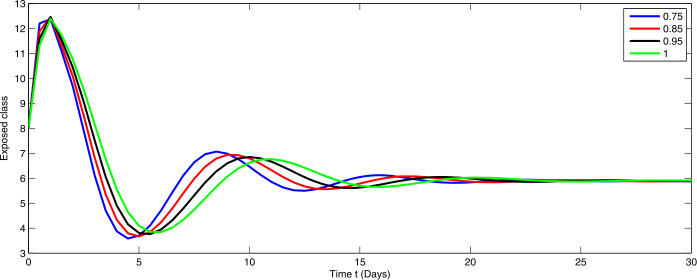
Graphical representation of the approximate solution $\mathcal{E}$ for the first ten terms at different fractional order

**Figure 2:**
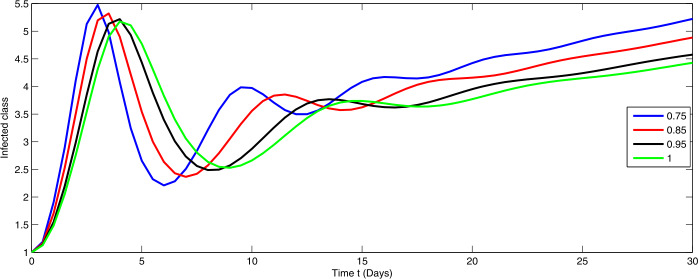
Graphical representation of the approximate solution $\mathcal{I}$ for the first ten terms at different fractional order

**Figure 3:**
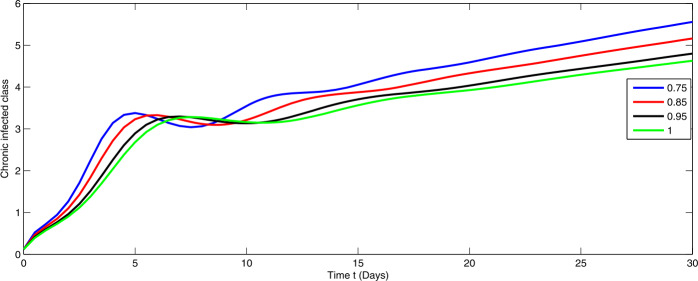
Graphical representation of the approximate solution $\mathcal{C}$ for the first ten terms at different fractional order

**Figure 4:**
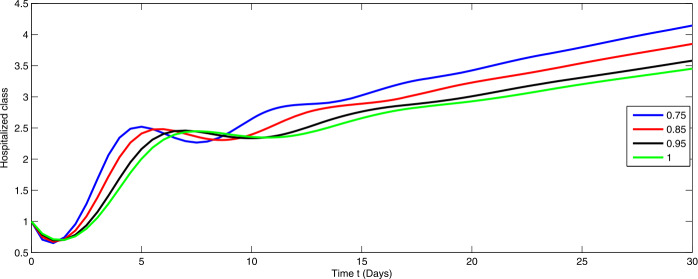
Graphical representation of the approximate solution $\mathcal{H}$ for the first ten terms at different fractional order

**Figure 5:**
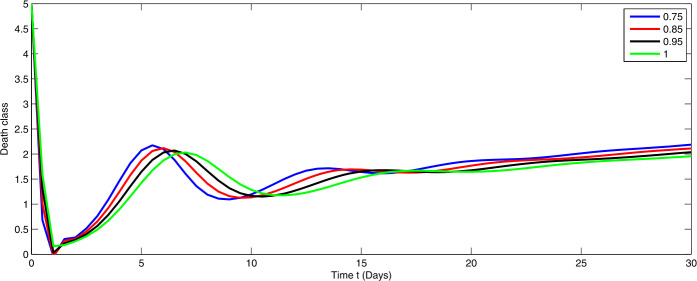
Graphical representation of the approximate solution $\mathcal{D}$ for the first ten terms at different fractional order

Here, we present the numerical simulation for the solution of the considered model (1.1)–(1.2) in the form of an infinite series as given in (4.8). Here the time is in days. The numerical values of the parameters utilized in the simulation are designated in Table 1. The graphical representations of the numerical solution of compartment $\mathcal{E}(t)$, $\mathcal{I}(t)$, $\mathcal{C}(t)$, $\mathcal{H}(t)$, $\mathcal{D}(t)$ with a various fractional order values $\theta =0.75, 0.85, 0.95, 1.0$ of the proposed model (1.1)–(1.2) are shown in Figs. 1–5, respectively. We consider the initial values $\mathcal{E}(0)=8 $, $\mathcal{I}(0)=1 $, $\mathcal{C}(0)=0.12 $, $\mathcal{H}(0)=1$, $\mathcal{D}(0)=5$.

**Table 1:** The physical interpretation of the parameters and numerical values

Parameters	Numerical values [45]
$\mathcal{B}$	0.80
*δ*	0.01
$\alpha _{1}$	0.55
$\alpha _{2}$	0.40
$\alpha _{3}$	0.60
$\alpha _{4}$	0.80
$\alpha _{5}$	0.34
$\alpha _{6}$	0.30
$\alpha _{7}$	0.35
$\alpha _{8}$	0.30
$\alpha _{9}$	0.35
$\alpha _{10}$	0.32

From Figs. 1–5, one can say that a huge population of exposed individuals becomes infected within a month. The decline in uninfected population is represented via different fractional order by taking the first ten terms. It is faster at smaller fractional order and slower at greater order. The decay occurs in the uninfected class under different fractional orders which is faster at lower fractional value by taking the first ten terms of the approximate solution in Fig. 1. As a result the exposed class will go up at a different rate due to the fractional order derivative as given in Fig. 2. This is because more people are exposed to infection. The infected class also goes on increasing. Here the growth is slow at lower fractional order as compared to higher order as in Fig. 3. Furthermore, the critically infected cases and hospitalization cases are also increasing as in Fig. 4. From Fig. 5, the death class leads to a fluctuation, maybe due to better care of infected people who recovered from the disease.

Furthermore, we investigate the dynamical behavior on increasing the values of the three parameters $\alpha _{i}$ ($i=1,2,3$), corresponding to integer order.

**Figure 6:**
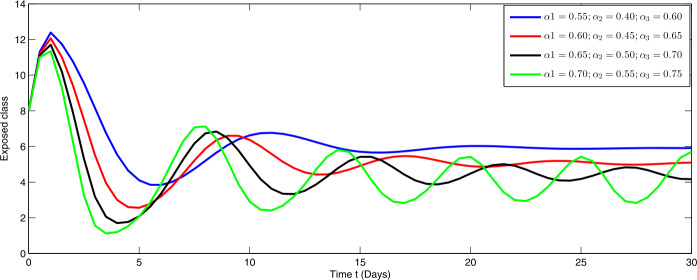
Dynamical behavior of $\mathcal{E}$ for different values of $\alpha _{i}$ ($i=1,2,3$) at integer order

**Figure 7:**
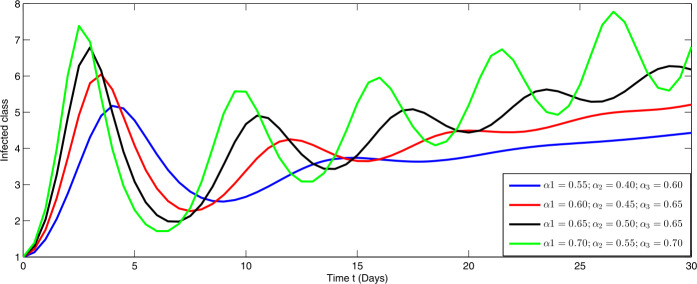
Dynamical behavior of $\mathcal{I}$ for different values of $\alpha _{i}$ ($i=1,2,3$) at integer order

**Figure 8:**
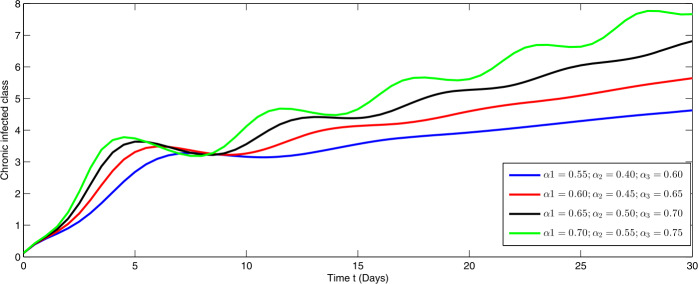
Dynamical behavior of $\mathcal{C}$ for different values of $\alpha _{i}$ ($i=1,2,3$) at integer order

**Figure 9:**
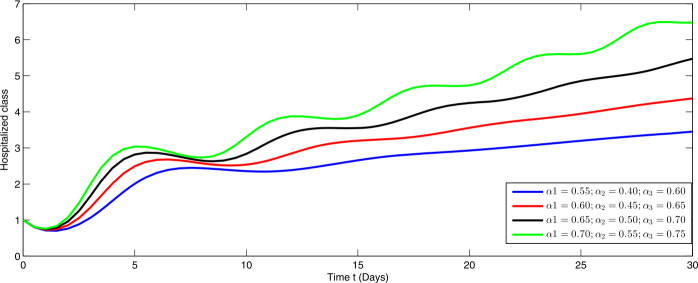
Dynamical behavior of $\mathcal{H}$ for different values of $\alpha _{i}$ ($i=1,2,3$) at integer order

**Figure 10:**
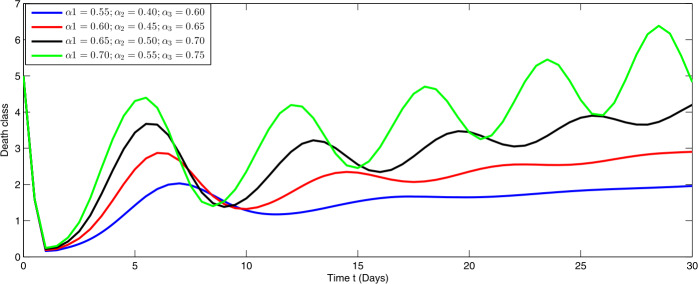
Dynamical behavior of $\mathcal{D}$ for different values of $\alpha _{i}$ ($i=1,2,3$) at integer order

In Figs. 6–10, we have presented the dynamical behavior at different values of the parameters $\alpha _{i}$ ($i=1,2,3$) corresponding to integer order. As we increase the values of $\alpha _{i}$ ($i=1,2,3$), the corresponding infection, hospitalization and chronic infection are increasing. Also the uncertain behavior has been observed on increasing the corresponding values of the aforesaid three parameters. Here we use a nonstandard finite difference scheme for the illustration of the dynamics at given values of the parameters.

## Conclusion

First of all, it is necessary that corresponding to a real-world problem the model one built would exist. This question should be guaranteed and in this regard the fixed point approach is a powerful analysis which gives proper information about the existence of such model. On the other hand, using a nonsingular derivative of fractional order for real-world problems is a new field in the last few years. Such investigations have been proved to get significant information about the global dynamics of an infectious disease. Furthermore, treating such a type of model by the Laplace Adomian decomposition method is another best way to handle an approximate solution of such a type of problems. For the Caputo–Fabrizo case, this concept has been very rarely adopted. The mentioned techniques omit discretization of data and need no collocation to control the method. Therefore, with the help of Picard’s iterative methods, we have successfully established a qualitative theory for a five compartment model of COVID-19 with Caputo–Fabrizo fractional order derivatives. Furthermore, some approximate analytical results have been developed via the Laplace Adomian decomposition method. The concerned solution has been presented via graphs for some numerical values. The fractional order derivative provides some more details of the transmission dynamics of the proposed model. In the future the concerned analysis can be extended to other mathematical models of infectious diseases.

## Data Availability

Not applicable.

## References

[1] Jeffrey S.K., Kenneth M. (2005). History and recent advances in coronavirus discovery. Pediatr. Infect. Dis. J..

[2] Feng Z., Castillo-Chavez C., Capurro A.F. (2000). A model for tuberculosis with exogenous reinfection. Theor. Popul. Biol..

[3] Kumar S., Kumar R., Singh J., Nisar K.S., Kumar D. (2020). An efficient numerical scheme for fractional model of HIV-1 infection of CD4+ T-cells with the effect of antiviral drug therapy. Alex. Eng. J..

[4] Ndairou F., Area I., Nieto J.J., Torres D.F. (2020). Mathematical modeling of COVID-19 transmission dynamics with a case study of Wuhan. Chaos Solitons Fractals.

[5] Syafruddin S., Noorani M.S.M. (2011). SEIR model for transmission of Dengue fever in Selangor Malaysia. Int. J. Mod. Phys. Conf. Ser..

[6] Tahir M., Shah I.S.A., Zaman G., Khan T. (2018). Prevention strategies for mathematical model MERS-corona virus with stability analysis and optimal control. J. Nanosc. Nanotechnol. Appl..

[7] Cao J., Jiang X., Zhao B. (2020). Mathematical modeling and epidemic prediction of COVID-19 and its significance to epidemic prevention and control measures. J. Biomed. Res. Innov..

[8] Chowell G., Blumberg S., Simonsen L., Miller M.A., Viboud C. (2014). Synthesizing data and models for the spread of MERS-CoV, 2013: key role of index cases and hospital transmission. Epidemics.

[9] Den V., Driessche P., Watmough J. (2002). Reproduction numbers and sub-threshold endemic equilibria for compartmental models of disease transmission. Math. Biosci..

[10] Fanelli D., Piazza F. (2020). Analysis and forecast of COVID-19 spreading in China, Italy and France. Chaos Solitons Fractals.

[11] Jung S.M., Akhmetzhanov A.R., Hayashi K., Linton N.M., Yang Y., Yuan B., Nishiura H. (2020). Real-time estimation of the risk of death from novel coronavirus (COVID-19) infection: inference using exported cases. J. Clin. Med..

[12] Kim Y., Lee S., Chu C., Choe S., Hong S., Shin Y. (2016). The characteristics of Middle Eastern respiratory syndrome coronavirus transmission dynamics in South Korea. Osong Public Health Res. Perspect..

[13] Lin Q., Zhao S., Gao D., Lou Y., Yang S., Musa S.S., Wang M., Cai Y., Wang W., Yang L., He D. (2020). A conceptual model for the coronavirus disease 2019 (COVID-19) outbreak in Wuhan, China with individual reaction and governmental action. Int. J. Infect. Dis..

[14] Kilbas A.A., Shrivastava H.M., Trujillo J.J. (2006). Theory and Applications of Fractional Differential Equations.

[15] Rihan F.A., Al-Mdallal Q.M., AlSakaji H.J., Hashish A. (2019). A fractional-order epidemic model with time-delay and nonlinear incidence rate. Chaos Solitons Fractals.

[16] Shah K., Alqudah M.A., Jarad F., Abdeljawad T. (2020). Semi-analytical study of Pine Wilt disease model with convex rate under Caputo–Fabrizio fractional order derivative. Chaos Solitons Fractals.

[17] Abdeljawad T., Al-Mdallal Q.M., Jarad F. (2019). Fractional logistic models in the frame of fractional operators generated by conformable derivatives. Chaos Solitons Fractals.

[18] Ivorra B., Ferrndez M.R., Vela-Pérez M., Ramos A.M. (2020). Mathematical modeling of the spread of the coronavirus disease 2019 (COVID-19) taking into account the undetected infections. The case of China. Commun. Nonlinear Sci. Numer. Simul..

[19] Shah, K., Abdeljawad, T., Mahariq, I., Jarad, F.: Qualitative analysis of a mathematical model in the time of COVID-19. BioMed Res. Int. 2020 (2020) doi.10.1155/2020/5098598 10.1155/2020/5098598 PMC7273369 32596319

[20] Ali, G., Nazir, G., Shah, K., Li, Y.: Existence theory and novel iterative method for dynamical system of infectious diseases. Discrete Dyn. Nat. Soc. 2020 (2020) doi.10.1155/2020/8709393

[21] Baleanu D., Aydogn S.M., Mohammadi H., Rezapour S. (2020). On modelling of epidemic childhood diseases with the Caputo–Fabrizio derivative by using the Laplace Adomian decomposition method. Alex. Eng. J..

[22] Baleanu D., Mohammadi H., Rezapour S. (2020). A fractional differential equation model for the COVID-19 transmission by using the Caputo–Fabrizio derivative. Adv. Differ. Equ..

[23] Chen T.M., Rui J., Wang Q.P., Cui J.A., Yin L. (2020). A mathematical model for simulating the phase-based transmissibility of a novel coronavirus. Infect. Dis. Poverty.

[24] Maier B.F., Brockmann D. (2020). Effective containment explains subexponential growth in recent confirmed COVID-19 cases in China. Science.

[25] Abdo M.S., Shah K., Wahash H.A., Panchal S.K. (2020). On a comprehensive model of the novel Coronavirus (COVID-19) under Mittag-Leffler derivative. Chaos Solitons Fractals.

[26] Abdulwasaa M.A., Abdo M.S., Shah K., Nofal T.A., Panchal S.K., Kawale S.V., Abdel-Aty A.H. (2020). Fractal-fractional mathematical modeling and forecasting of new cases and deaths of COVID-19 epidemic outbreaks in India. Results Phys..

[27] Redhwan S.S., Abdo M.S., Shah K., Abdeljawad T., Dawood S., Abdo H.A., Shaikh S.L. (2020). Mathematical modeling for the outbreak of the coronavirus (COVID-19) under fractional nonlocal operator. Results Phys..

[28] Trilla A. (2020). One world, one health: the novel coronavirus COVID-19 epidemic. Med. Clin. (Barc.).

[29] Wong G., Liu W., Liu Y., Zhou B., Bi Y., Gao G.F. (2015). MERS, SARS, and Ebola: the role of super-spreaders in infectious disease. Cell Host Microbe.

[30] Borah M.J., Hazarika B., Panda S.K., Nieto J.J. (2020). Examining the correlation between the weather conditions and COVID-19 pandemic in India: a mathematical evidence. Results Phys..

[31] Panda S.K. (2020). Applying fixed point methods and fractional operators in the modelling of novel coronavirus 2019-nCoV/SARS-CoV-2. Results Phys..

[32] Atangana A. (2020). Modelling the spread of COVID-19 with new fractal-fractional operators: can the lockdown save mankind before vaccination?. Chaos Solitons Fractals.

[33] Khan M.A., Atangana A. (2020). Modeling the dynamics of novel coronavirus (2019-nCov) with fractional derivative. Alex. Eng. J..

[34] Naik P.A., Yavuz M., Qureshi S., Zu J., Townley S. (2020). Modeling and analysis of COVID-19 epidemics with treatment in fractional derivatives using real data from Pakistan. Eur. Phys. J. Plus.

[35] Memon Z., Qureshi S., Memon B.R. (2021). Assessing the role of quarantine and isolation as control strategies for COVID-19 outbreak: a case study. Chaos Solitons Fractals.

[36] Atangana E., Atangana A. (2020). Facemasks simple but powerful weapons to protect against COVID-19 spread: can they have sides effects?. Results Phys..

[37] Caputo M., Fabrizio M. (2015). A new definition of fractional derivative without singular kernel. Prog. Fract. Differ. Appl..

[38] Bas E., Acay B., Ozarslan R. (2019). Fractional models with singular and non-singular kernels for energy efficient buildings. Chaos, Interdiscip. J. Nonlinear Sci..

[39] Abdon A., Baleanu D. (2017). Caputo–Fabrizio derivative applied to groundwater flow within confined aquifer. J. Eng. Mech..

[40] Atangana A., Alkahtani B.S.T. (2015). Analysis of the Keller–Segel model with a fractional derivative without singular kernel. Entropy.

[41] Khan M.A., Hammouch Z., Baleanu D. (2019). Modeling the dynamics of hepatitis E via the Caputo–Fabrizio derivative. Math. Model. Nat. Phenom..

[42] Koca I. (2018). Analysis of rubella disease model with non-local and non-singular fractional derivatives. Int. J. Optim. Control Theor. Appl..

[43] Hussain A., Baleanu D., Adeel M. (2020). Existence of solution and stability for the fractional order novel coronavirus (nCoV-2019) model. Adv. Differ. Equ..

[44] Thabet S.T.M., Abdo M.S., Shah K., Abdeljawad T. (2020). Study of transmission dynamics of COVID-19 mathematical model under ABC fractional order derivative. Results Phys..

[45] Shah N.H., Suthar A.H., Jayswal E.N. (2020). Control strategies to curtail transmission of COVID-19. Int. J. Math. Math. Sci..

[46] Losada J., Nieto J.J. (2015). Properties of a new fractional derivative without singular kernel. Prog. Fract. Differ. Appl..

[47] Lyons R., Vatsala A., Chiquet R. (2017). Picard’s iterative method for Caputo fractional differential equations with numerical results. Mathematics.

[48] Rida S.Z., Arafa A.A.M., Gaber Y.A. (2016). Solution of the fractional epidemic model by L-ADM. J. Fract. Calc. Appl..

[49] Shah K., Abdeljawad T., Jarad F. (2020). On a nonlinear fractional order model of Dengue fever disease under Caputo–Fabrizio derivative. Alex. Eng. J..

